# Precise Calculation of a Bond Percolation Transition and Survival Rates of Nodes in a Complex Network

**DOI:** 10.1371/journal.pone.0119979

**Published:** 2015-04-17

**Authors:** Hirokazu Kawamoto, Hideki Takayasu, Henrik Jeldtoft Jensen, Misako Takayasu

**Affiliations:** 1 Department of Computational Intelligence and Systems Science, Interdisciplinary Graduate School of Science and Engineering, Tokyo Institute of Technology, Midori-ku, Yokohama, Japan; 2 Sony Computer Science Laboratories, Shinagawa-ku, Tokyo, Japan; 3 Meiji Institute for Advanced Study of Mathematical Sciences, Meiji University, Nakano-ku, Tokyo, Japan; 4 Department of Mathematics and Complexity & Networks Group, Imperial College, London, United Kingdom; Beihang University, CHINA

## Abstract

Through precise numerical analysis, we reveal a new type of universal loopless percolation transition in randomly removed complex networks. As an example of a real-world network, we apply our analysis to a business relation network consisting of approximately 3,000,000 links among 300,000 firms and observe the transition with critical exponents close to the mean-field values taking into account the finite size effect. We focus on the largest cluster at the critical point, and introduce survival probability as a new measure characterizing the robustness of each node. We also discuss the relation between survival probability and k-shell decomposition.

## Introduction

Percolation theory is a pillar of statistical physics that provides a basic understanding of transitions by studying macroscopic connectivity in a system as its elements are randomly removed [1]. As connectivity is a fundamental general property, we can apply percolation theory to a variety of fields such as electrical conduction [2], fracture mechanics [3], flow of fluids in porous materials [4], spread of epidemics [5], Internet traffic congestion [6], and the flow of sea ice and blood [7, 8]. It is well-known that the critical point of a system, at which global connectivity is suddenly lost, depends on the details of the model of the system at hand. However, values of critical exponents for models in Euclidean space are fairly uniform because they depend only on spatial dimension [1, 9, 10].

Network models have recently garnered considerable attention from physicists because these models are defined out of the Euclidean space and have led to many new insights [11–13]. Network models consist of nodes and links, where links connect interacting pairs of nodes. Such a framework is suitable for the description of various phenomena such as protein-protein interaction [14], information flow among servers on the Internet [15], human relations based on telephone communication [16], money flow among banks [17, 18], and transaction networks of businesses [13, 19, 20]. A feature common to these real-world networks is that they are scale-free, i.e., their degree distribution follows a power law, at least asymptotically. This feature implies that each of these networks is composed of several large nodes with thousands of links, many intermediate nodes and a majority of very small nodes with few links [12].

The study of percolation processes in such complex networks is important given the fragility of the systems being modeled [21–25]. It is well-known that scale-free networks lose connectivity at high density if nodes are removed in descending order of the degree, showing typical transition behavior [21]. In random removal of nodes or links, however, scale-free networks maintain connectivity, even at very low density, and no observations of a percolation transition have been reported so far. In this study, through precise numerical calculation of a real world example, we prove the existence of percolation transition in complex networks in random removal of links when the network density is very low but non-zero.

In Section 2, we introduce data gathered from approximately 600,000 Japanese firms and describe its basic statistical properties. We present a precise observation of percolation transition in networks in Section 3. Section 4 discusses finite size effect is discussed for the clipped networks. Section 5 reveals a structural change of network. We show finite size scaling around the transition in Section 6. Section 7 discusses percolation transition of Erdös-Rényi graph. In Section 8, we propose an index to measure the robustness of nodes and their survival probability, and compare it with k-shell decomposition. Finally, we discuss our findings and conclude in Section 9.

## Business relation network

**Fig 1:**
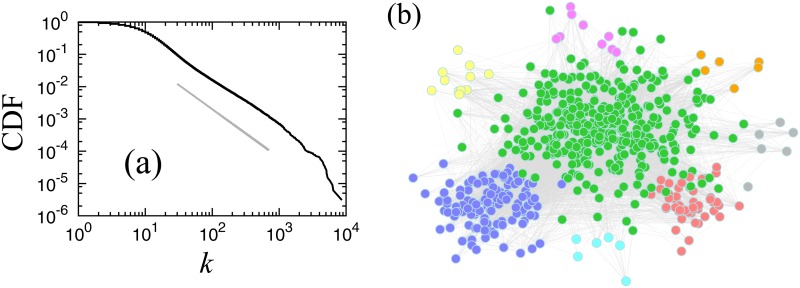
**(a) Cumulative distribution *F*(≥ *k*) of link numbers in log-log plot**. The guideline (solid line) shows the slope of a power law with the cumulative exponent, 1.5. This distribution follows a power law on a large scale, *F*(≥ *k*) ∝ *k* ^−1.5^. **(b) Japanese business relation network for firms with more than 1,000 links**. Hokkaido region (orange), Tohoku region (grey), Kanto region (including Tokyo) (green), Chubu region (including Nagoya) (red), Kansai region (including Osaka) (purple), Chugoku region (pink), Shikoku region (skyblue), Kyushu-Okinawa region (yellow).

Network data for our project is provided by TEIKOKU DATABANK, Ltd., a Japanese credit research company in Tokyo. This data contains information about the direction of money flow among businesses in Japan in 2011, defining the business relation network. From this network, we select the largest strongly connected component (LSCC), where LSCC is the largest part of the network in which every node is connected to every other node by at least one unidirectional path [26]. Our LSCC network consists of 327,721 nodes, and we connect trading pairs directly through undirected links for percolation analysis. The LSCC has 2,960,370 links, and the link number distribution of this network is approximated by a power law for large link numbers with a cumulative exponent of 1.5, as shown in Fig 1(a) and in Eq (1).

$$\begin{array}{c} F\left(\geq k\right)\propto k^{-1.5} \end{array}$$ (1)

Here, *F*(≥ *k*) denotes the cumulative distribution function of link numbers *k*. Similar power laws have been reported for business relation networks based on different data sources [27–30]. Not only is this network scale-free, it also has a small-world characteristic that the most likely distance between a pair of firms is 5 links; whereas, the longest pair distance is 21 links. In Fig 1(b), we plot the network structure for firms with more than 1,000 links.

## Observation of percolation transition

We provide the numerical computation of percolation analysis as follows. For given network clusters, we first categorize all links into bridge links and loop links. A bridge link is a link that, if removed, divides a network cluster into two clusters or a cluster and an isolated node, which is a cluster with link size 0 and node size 1. Loop links are links whose removal does not alter the connectivity of the network. We randomly choose a link and remove it from the network. If it is a bridge link, the network cluster is divided into two clusters, and we measure the sizes of each according to the number of links in each. If the link removed is a loop link, the size of the cluster is simply reduced by 1. After removal, we calculate the largest cluster size, the average cluster size and the cluster size distribution, which are the basic quantities in percolation theory. We repeatedly apply the process of categorization of bridges and loops, random link removal and calculation of basic quantities to the LSCC network until all links have been removed. This process constitutes one realization of our numerical simulation. We perform at most 10,000 realizations using different random number seeds for the calculation of averages and estimation of the survival rate.

**Fig 2:**
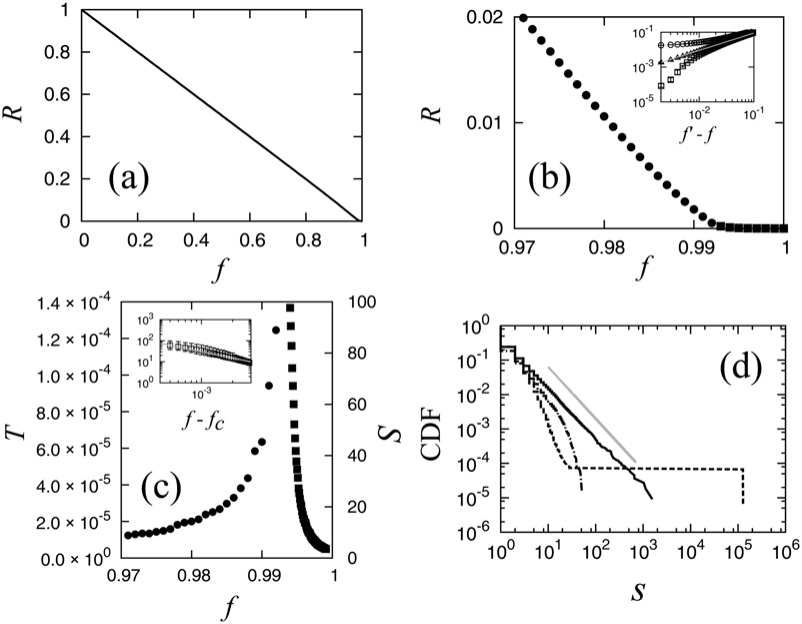
**(a) Order parameter *R* in the whole range of *f*. (b) Order parameter *R* in the range of *f* between 0.97 and 1.00**. Circles and squares specify values below and above the critical point, respectively. (Inset) Log-log plots of *R* vs. *f*′−*f*. *f*′ < *f*_c_ (circles), *f*′ = *f*_c_ (triangles), and *f*′ > *f*_c_ (squares). The grey guideline shows the power law with critical exponent *β* = 1.0. Error bars estimated by the interquartile range (IQR) from 100 trials using different random number seeds are plotted (all error bars are within the size of plotted squares.). **(c) Normalized second largest cluster size *T* below the critical point (circles), and the average cluster size *S* above the critical point (squares)**. (Inset) Average cluster size *S* and *f*−*f*_c_ in log-log scale. The grey guideline shows the slope for the critical exponent *γ* = 1.0. **(d) Cumulative cluster size distributions in log-log scale**. The dot-dash, bold and dash lines show values below, at, and above the critical point, respectively. The guideline shows a slope of 1.5, corresponding to the critical exponent *τ* = 2.5. The results are a superposition of 10 trials.

The order parameter *R* is defined as the ratio of the link number of the largest cluster to that of the original LSCC, and the control parameter *f* is defined as the ratio of the number of removed links to the total number of links in the original LSCC. In Fig 2(a) the value of *R* is plotted as a function of *f*. It appears that *R* decays linearly and moves toward 0 at *f* = 1. However, by zooming into the range of *f* between 0.97 and 1.00, as shown in Fig 2(b), we find that the order parameter *R* becomes approximately 0 at a non-trivial value of *f*, indicating the existence of a percolation transition. To calculate the position of the transition, we determine the value of *f* for which the second largest cluster becomes the largest by the removal of a link that divides the largest cluster into two smaller clusters. This value of *f* is denoted by *f*_*c*_, and it is the critical point. This method is similar to that of estimating the critical point by observing the ratio of the size of the second largest cluster to the size of the largest cluster [31]. We repeat the simulation 100 times with different random numbers and calculate the critical point as *f*_c_ = 0.994.

The behavior of *R* around the critical point is plotted in log-log scale in the inset of Fig 2(b). Eq (2) represents a power law decay of *R* at the transition point, and is confirmed for the critical exponent *β* = 1.0. We tested various candidate values for the critical point, and verified that the value *f*_c_ = 0.994 renders the plots straight in the widest range.

$$\begin{array}{c} R\propto {\left(f_{c}-f\right)}^{\beta } \end{array}$$ (2)

The next standard characterization of percolation transition is divergence of the average cluster size, *S*, which is defined as ⟨*s* ^2^⟩/⟨*s*⟩, where *s* is the size of a cluster and ⟨⋅⟩ denotes the average over all clusters for all realizations. In the range of *f* above *f*_c_ this divergence is confirmed as shown in Fig 2(c), and the critical exponent *γ*, which is defined by Eq (3), is roughly estimated to be 1.0 from the inset of Fig 2(c).

$$\begin{array}{c} S\propto {\left(f-f_{c}\right)}^{-\gamma } \end{array}$$ (3)

In the range of *f* below the critical point it is difficult to observe this divergence directly as we have to remove the largest cluster for the calculation of *S*. Instead of the conventional average cluster size, we observe the average size of the second largest cluster in this range because this quantity most clearly characterizes the tendency of divergence of fluctuation in cluster size near the critical point as shown by the red triangles in Fig 2(c). We confirm that this characteristic cluster size also shows divergence at the critical point just like ordinary percolation transitions.

As a third method for the verification of percolation transition we observe the cumulative distribution of cluster size *P*(≥ *s*) in log-log scale as shown in Fig 2(d). We define the cumulative cluster size distribution as follows:

$$\begin{array}{c} P\left(\geq s\right)=\sum_{s^{\prime }=s}^{s_{m}}P\left(s^{\prime }\right) \end{array}$$ (4)

where *P*(*s*) is the probability density function (PDF) and *s*_m_ denotes the largest cluster size. The distribution decays quickly above the critical point, *f* > *f*_c_, obeys a power law at the critical point *f* = *f*_c_, and has a huge cluster below the critical point, *f* < *f*_c_, as shown by the hill of the cumulative distribution. The slope of the guideline in Fig 2(d) shows the power exponent value of 1.5 and critical exponent value *τ*, which is defined as in Eq (5), and is calculated to be 2.5.

$$\begin{array}{c} P\left(s\right)\propto s^{-\tau } \end{array}$$ (5)

In sum, the obtained values of critical exponents *β* = 1.0, *γ* = 1.0 and *τ* = 2.5 fulfill the scaling relationship described in Eq (6) below, and fit well with the values of the mean-field critical exponents, which are exact for a Bethe lattice with no loop or for Euclidean space with spatial dimension higher than 6 [32].

$$\begin{array}{c} \tau -2=\frac{\beta }{\beta +\gamma } \end{array}$$ (6)

## Finite size effect

Next, we consider finite size dependence. For this purpose we prepare some regional parts of various sizes of business firm networks as we did in the case of Fig 1(a). We apply the same percolation analysis for these clipped networks.

**Fig 3:**
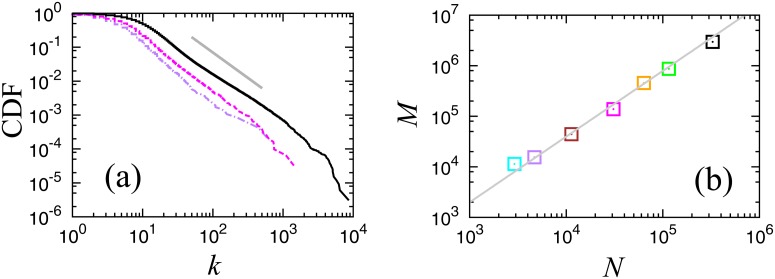
**(a) Cumulative distributions of link numbers in a log-log plot**. Nationwide (black solid line), Osaka prefecture (magenta dotted line), and Miyagi prefecture (purple dot-dash line). The guideline (grey thin line) shows the slope for a power law with the cumulative exponent, 1.5. **(b) Initial number of links *M* and the number of nodes *N* for seven networks of different sizes in a log-log plot**. The slope of the guideline shows the power exponent *ϕ* = 1.3. Nationwide (black), Kanto region (green), Tokyo prefecture (orange), Osaka prefecture (magenta), Fukuoka prefecture (brown), Miyagi prefecture (purple), and Kagoshima prefecture (cyan).

In Fig 3(a), cumulative link number distributions are plotted for two regional parts of the whole network extracted by using the information regarding the location of the firms, Osaka prefecture (30,766 firms and 138,425 links), and Miyagi prefecture (4,686 firms and 15,443 links). Comparing with the distribution for the whole country, we find that the distributions shift depending on the system size, following the same power law implying that the business relation network has a scale-free property for link numbers larger than 10.

In order to observe the finite size effect more accurately we focus on the number of nodes *N* and the link number *M* at *f* = 0, the initial networks with no random removal. In Fig 3(b), *M* is plotted against *N* in a log-log scale, a non-trivial scaling law, *M* ∝ *N* ^*ϕ*^ is confirmed with the power exponent *ϕ* about 1.3. It is trivial that the value of *ϕ* is 1.0 for Euclidean lattices, and in artificial scale-free networks following the power law link number distribution in the whole range, this exponent *ϕ* is 1.0 theoretically, if the exponent of the cumulative link number distribution is larger than 1. As the mean link number is given by 2*M*/*N*, the exponent *ϕ* > 1 implies that the mean link number diverges for a large system size limit. In a real business firm network, this effect is realized by the shift of power law of the link number distribution, as seen in Fig 3(a), implying that the geographically long range direct interaction among business firms in different regions is non-negligible.

**Fig 4:**
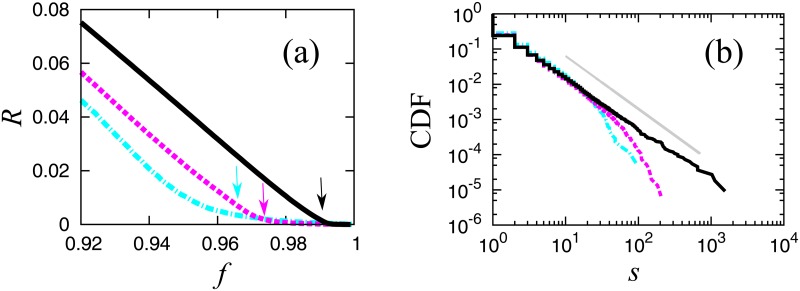
**(a) Order parameter *R* for three networks: Nationwide (black solid line), Osaka pre-fecture (magenta dashed line), and Kagoshima prefecture (cyan chain line)**. The arrows indicate the corresponding critical points. **(b) Cumulative cluster size distributions at the critical points in a log-log scale for the three cases shown in (a)**.

In Fig 4(a) and 4(b), we show the order parameters and the cumulative cluster size distributions at the critical point for two smaller size networks plotted with the case for the whole system. In both cases, the transition behaviors are less clear for smaller systems; however, we can confirm that the critical exponents are independent of the system size.

## Structural change of network

**Fig 5:**
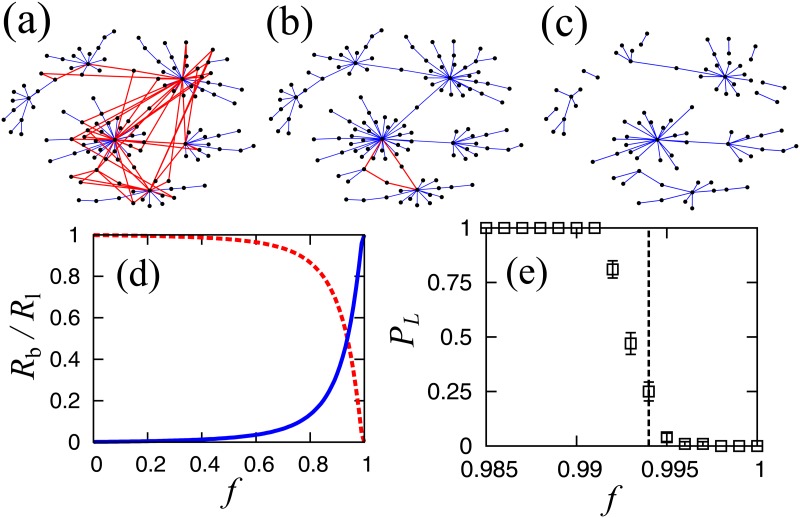
**Examples of typical clusters; (a) *f* < *f*_c_, (b) *f* = *f*_c_ and (c) *f* > *f*_c_**. Bridge links and loop links are shown in blue and red, respectively. **(d) Ratio of bridge links (blue line) and loop links (broken red line) in the largest cluster. (e) Probability that the largest cluster has a loop**. The broken black line shows the critical point. Results are estimated for 100 trials.

The mean-field approximation of percolation theory is based on the assumption of the loopless tree structure, and its applicability is directly checked numerically. Parts of typical network configurations are shown in Fig 5(a), 5(b) and 5(c) for cases where *f* < *f*_c_, *f* = *f*_c_ and *f* > *f*_c_, respectively. We see in these figures that a large number of loop links, drawn in red, are below the transition point, and that all links above the critical point are bridge links. In Fig 5(d), we plot the ratio of the number of bridge links *R*_b_ to that of loop links *R*_l_ = 1−*R*_b_ as a function of *f* for the original LSCC. For very small values of *f*, almost all links are loop links, and *R*_l_ decreases gradually before rapidly decaying to 0 for values of *f* close to 1. In order to observe the behavior of the system around *f* = 1 in detail, we calculate the probability *P*_L_ that the largest cluster has at least one loop by repeating the simulation 100 times, as shown in Fig 5(e). We show that this probability abruptly decreases to 0 around the transition point, i.e., the percolation transition point is also the loop-less transition point. Around the critical point, the contribution of loops is very small. Therefore, the mean-field approximation becomes exact such that the critical exponents belong to the mean-field university class.

**Fig 6:**
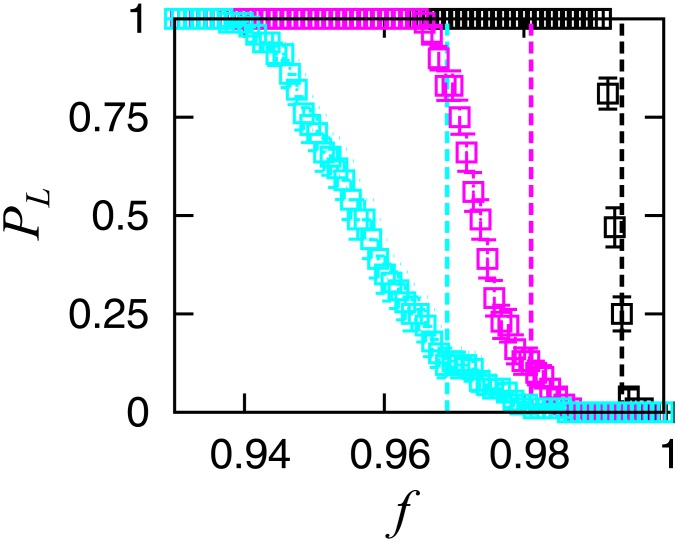
Size dependence of the probability that the largest cluster has a loop for the three cases shown in Fig 4. The dotted lines indicate the corresponding critical points.

We also confirm size dependency of this structural change. We plot the probability that the largest cluster has at least a loop for the three cases in Fig 6. The loops disappear around each transition point, independent of network size.

## Finite size scaling

**Fig 7:**
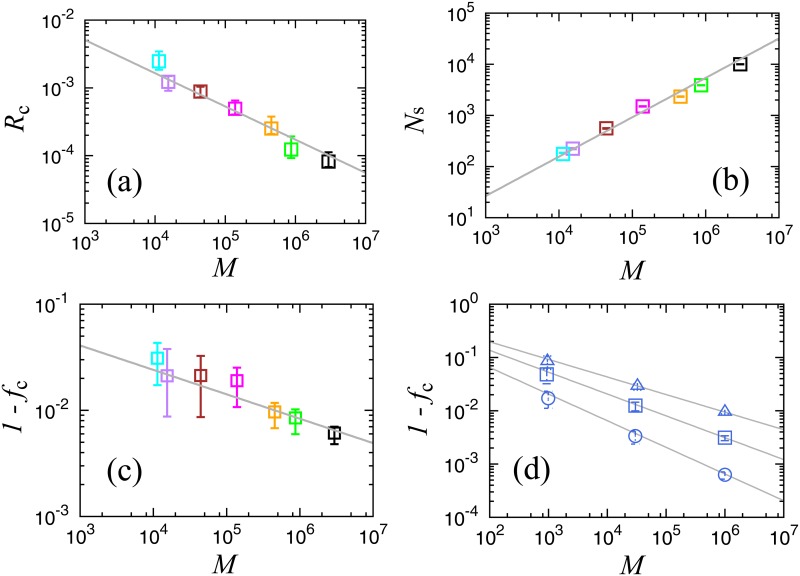
**(a) Normalized size of the largest cluster *R*_*c*_ at the critical point for each network shown in Fig 3(b)**. The line shows the slope for *R*_*c*_ ∝ *M* ^−*δ*^, *δ* = 0.50. Colors are the same as in Fig 3(b). **(b) Number of clusters at the critical point for each network shown in Fig 3(b)**. The line shows the slope for *N*_*s*_ ∝ *M* ^*ρ*^, *ρ* = 0.77. **(c) Critical link density as a function of M for each network shown in Fig 3(b)**. The line shows the slope for 1−*f*_*c*_ ∝ *M* ^−*ε*^, *ε* = 0.23. The number of trials ranges from 1,000 to 100,000, depending on convergence speed for each network. The error bars indicate the interquartile range (IQR). **(d) Critical link density on Erdös-Rényi graph (ER-graph), *ϕ* = 1.5 (triangles), *ϕ* = 1.7 (squares), and *ϕ* = 2.0 (circles)**. The number of trials ranges from 1,000 to 100,000, depending on convergence speed for each network.

We introduce three more scaling relations for characterization of the critical point in the initial size of the clipped networks, *M*. In Fig 7(a) we observe *R*_c_, the value of the order parameter at the critical point, which is given by the largest cluster’s link number divided by the initial link numbers, *M*. From this log-log plot we find that the scaling relation, *R*_c_ ∝ *M* ^−*δ*^, holds for *δ* = 0.50. In Fig 7(b), the number of clusters at the critical point for each clipped network is plotted as a function of *M*, and we confirm another power law, *N*_*s*_ ∝ *M* ^*ρ*^, where *ρ* is approximately 0.77. In Fig 7(c), the scaling relation for the critical link density, 1−*f*_c_ ∝ *M* ^−*ε*^, holds for *ε* about 0.23. This relation demonstrates that the critical link density converges to 0 in the large system limit; therefore, we may call this the sparse graph limit transition.

We now theoretically derive the relation among scaling exponents. Let *n*, *n*_m_, and *Q*(*n*) denote the node size of a cluster, largest cluster’s node size and node size PDF of clusters, respectively. Considering the probability of existence of the largest cluster when there are totally *N*_*s*_ clusters, we expect the following relation to hold:

$$\begin{array}{c} \sum_{n=n_{m}}^{\infty }Q\left(n\right)\approx \frac{1}{N_{s}} \end{array}$$ (7)

By the conservation of node numbers we also require the following general relation:

$$\begin{array}{c} N_{s}\sum_{n=1}^{n_{m}}nQ\left(n\right)\approx N \end{array}$$ (8)

At the critical point we can assume that the numbers of nodes and links are nearly equal, *n* ≈ *s*, as the clusters are almost loop-less; therefore, *Q*(*n*) is replaced by the power law, *P*(*s*) ∝ *s* ^−*τ*^. Then, approximating the summation by integral, we obtain the scaling laws, $s_{\text{m}}^{\tau -1}\propto N$ and *N*_*s*_ ∝ *N* from Eqs (7) and (8), respectively. As *R*_c_ is defined by *s*_m_/*M* and *M* ∝ *N* ^*ϕ*^, also 1−*f*_c_ = (*N*−*N*_*s*_)/*M*, we have the following relations for *δ*, *ρ*, and *ε*, defined by *R*_c_ ∝ *M* ^−*δ*^, *N*_*s*_ ∝ *M* ^*ρ*^, and 1−*f*_c_ ∝ *M* ^−*ε*^

$$\begin{array}{c} \delta =1-\frac{1}{\Phi (\tau -1)},\rho =\frac{1}{\Phi },\epsilon =1-\frac{1}{\Phi } \end{array}$$ (9)

These relations are confirmed by introducing the observed values for *τ* and *ϕ*, *δ* = 0.49(0.50), *ρ* = 0.77(0.77) and *ε* = 0.23(0.23), where the numbers in parentheses show the directly observed values.

## Erdös-Rényi graph

We can also observe the sparse graph limit transition in a complete graph in which all nodes are directly connected. Let the number of nodes be *N*, then the initial link number *M* is given as *N*(*N*−1)/2, and hence, the exponent *ϕ* is 2. It is known that the percolation transition occurs and the critical value is characterized by 1−*f*_c_ = 1/*N*, namely, 1−*f*_c_ ∝ *M* ^−*ε*^ with *ε* = 1/2, which is consistent with the last equation of Eq (9) [33, 34]. The critical exponents for the complete graph take the same mean field values.

To confirm the previously described scaling relation with a theoretical model, we introduce percolation simulations for Erdös-Rényi graphs (ER-graphs). First, we prepare a set of ER-graphs that follow the relation, *M* ∝ *N* ^*ϕ*^. For a given node number *N*, we choose links randomly with the link density 2*M*/(*N*(*N*−1)), and create three series of ER-graphs, (*N*, *M*) = (200, 956), (2048, 32558), (20000, 1001507) for *ϕ* = 1.5, (*N*, *M*) = (116, 932), (860, 30039), (6761, 1001454) for *ϕ* = 1.7, (*N*, *M*) = (45, 990), (245, 29890), (1415, 1000405) for *ϕ* = 2.0. Here, ER-graphs with *ϕ* = 2.0 are complete networks. We regard these nine model networks as the initial networks, and we remove links randomly to estimate the critical points using our method of searching the point where the largest cluster size becomes roughly equivalent to the second largest cluster size. The number of trials for removal ranges from 1,000 to 100,000 times, depending on network size and corresponding to the precision of the real data simulation, as shown in Fig 7(c). In Fig 7(d), we confirm that the simulation and our theory are in good agreement for these three series, *ϕ* = 1.5,1.7, and 2.0.

**Fig 8:**
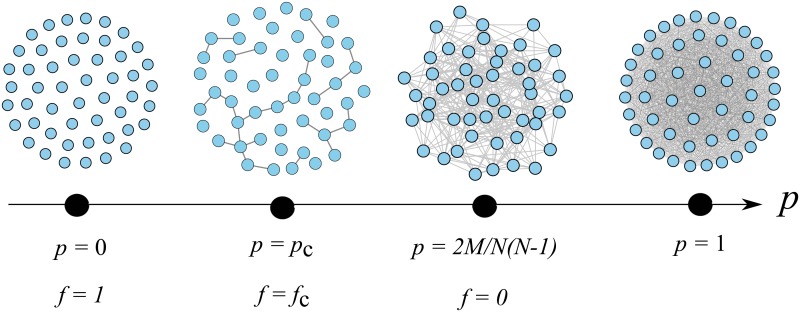
Schematic figures of the percolation process of a complete graph. In our simulation, an initial state (*f* = 0) is chosen as an ER-graph with a link density *p* between *p* = *p*_*c*_ and 1, and we consider removal process of links toward *p* = 0(*f* = 1).

In the previously described simulation, we regard the ER-graphs as the initial states (*f* = 0), in which the link density, *p*, is given by 2*M*/(*N*(*N*−1)), as schematically shown in Fig 8. In the theory of ER-graphs, the critical point of percolation phase transition is given by *p*_*c*_ = 1/*N* [33], and the relation between *f* and *p* is as follows:

$$\begin{array}{c} p=1-\frac{\frac{N(N-1)}{2}-M+m}{\frac{N(N-1)}{2}}=\frac{2M(1-f)}{N(N-1)} \end{array}$$ (10)

where *N*(*N*−1)/2−*M* is the difference of link numbers between the complete graph and initial ER-graph with M links, *m* is the number of links removed from the initial state (*f* = 0), and *f* = *m*/*M*. As the critical point of the ER-graph is given by *p*_c_ = 1/*N*, we have the following relation:

$$\begin{array}{c} 1-f_{c}=\frac{N-1}{2M} \end{array}$$ (11)

Under our finite size scaling assumption, *M* ∝ *N* ^*ϕ*^, we obtain the following equation:

$$\begin{array}{c} 1-f_{c}\propto M^{-1+1/\Phi } \end{array}$$ (12)

This equation is consistent with Eq (9). Therefore, we understand that the scaling relations, Eq (9), is a natural generalization of the percolation theory of ER-graphs to more general complex networks.

We expect that the sparse graph limit transition is a general property which is applicable to any complex networks that follow finite size scaling, *M* ∝ *N* ^*ϕ*^ with *ϕ* > 1, as the mean link number per node at the critical point is 1 and the link density at the critical point vanishes in the limit of *N* → ∞. Also, the fact that the critical cluster becomes a loop-less tree is expected to be a general property that makes the critical exponents given by the mean-field values.

## Survival rates of nodes

**Fig 9:**
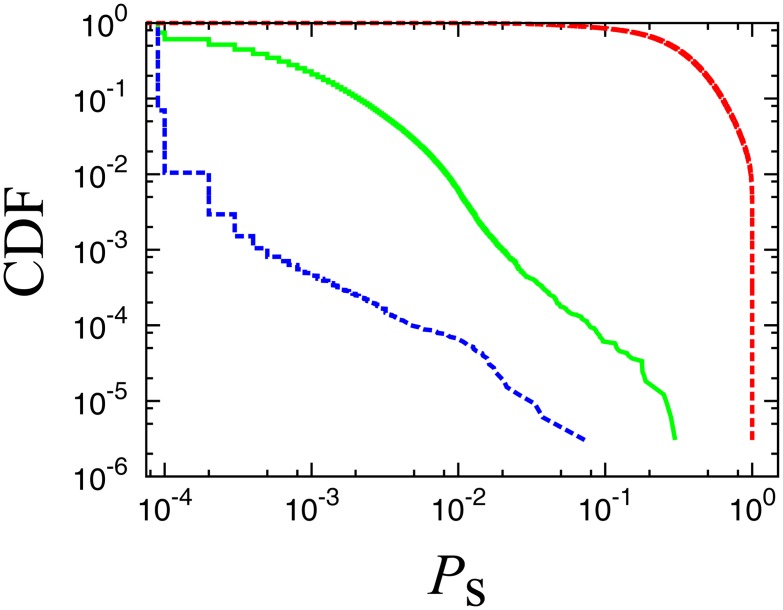
Cumulative distributions of the survival rates. The red, green and blue lines represent values below (*f* = 0.950), at (*f* = 0.994), and above (*f* = 0.9999) the critical point, respectively. The values of survival rates are distributed most widely at the critical point.

In this section, we calculate the survival rate, *P*_*s*_, of each node as a function of *f* by repeating multiple trials using different random numbers. We define the survival rate as the ratio of the number of trials, where the node belongs to the largest cluster, to the total number of trials. We perform 10,000 trials and calculate the survival rate function for each node. Fig 9 shows the distributions of survival rates for three values of *f*, i.e., *f* = 0.950 (below *f*_*c*_), *f* = 0.994 (at the critical point *f*_*c*_) and *f* = 0.9999 (above *f*_*c*_). Below the critical point (the broken red line), there are many nodes whose survival rates are very close to 1, and thus, we cannot characterize the differences among these nodes. Above the critical point (the broken blue line), many nodes (more than 90% of the total number of nodes) record survival rates less than the observation limit (10^−4^). At the critical point, the survival rates are most widely distributed, implying that the survival rate at the critical point can be a new measure of the robustness of nodes against random attacks.

**Fig 10:**
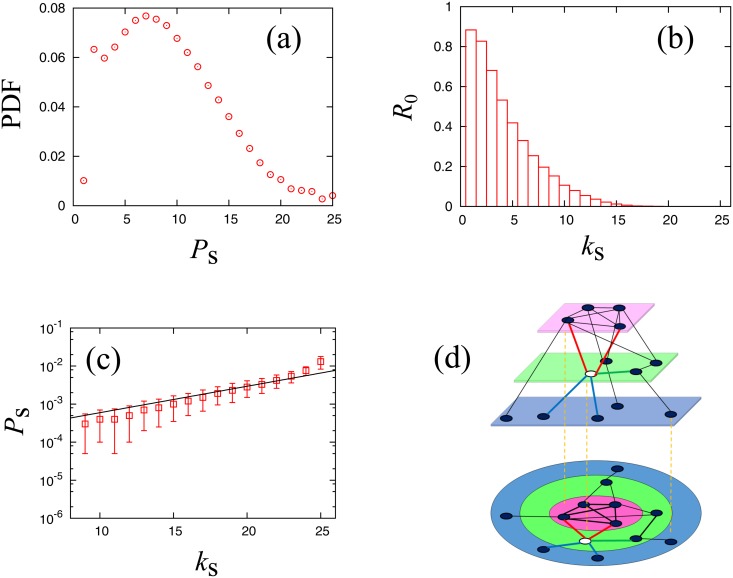
**(a) PDF of nodes belonging to the *k*_s_-th shell**. The total number of shells is 25 and the most populated shell is *k*_s_ = 7. **(b) Ratio of the number of nodes for each shell that did not survive the 10,000 trials. (c) Median of the survival rate for each shell for ranging from *k*_*s*_ ≥ 9**. Error bars are plotted using quartile deviation. The guideline shows *P*_*s*_ ∝ exp(*Bk*_*s*_) where *B* = 0.16. **(d) Schematic figure of the degree of decomposition in k-shell decomposition analysis**. Each plate shows the shell (*k*_*s*_ = 1 (blue); 2 (green); 3 (pink)). Focusing on the white node, the red links are oriented towards a higher shell, and their number is denoted by *k*_*u*_. The green links are oriented in the same shell, and their number is *k*_*m*_. The blue links are oriented to a lower shell, and their number is *k*_*d*_.

For a more detailed characterization of the structure of this business transaction network, we apply *k*-shell decomposition analysis [35] to the network to calculate the number of shells in the network and number of nodes in each shell. We define a *k*-shell as the set of nodes belonging to the *k*-core but not to the (*k*+1)-core, where k-core is defined by the maximal sub-graph having a minimal link number *k*. This decomposition characterizes the importance of nodes in a complex network structure. As a result, we find that the business relation network is decomposed into 25 shells. We assign an integer index *k*_s_ to each node that represents the shell number to which the node belongs. As shown in Fig 10(a), the distribution of shell numbers is maximum at *k*_s_ = 7, and there are 1,346 nodes with the largest index *k*_s_ = 25. The number of nodes at the periphery (*k*_s_ = 1) is very small because we extracted the LSCC from raw network data.

We count the number of nodes, for each shell, that were never part of the largest cluster at the critical point in the 10,000 trials, and calculate the ratio *R*_0_ by dividing it by the total number of nodes in the shell. Fig 10(b) shows that this value is large for small shell numbers. We note that the ratio *R*_0_ decreases for all shells when the number of trials is increased to lower the observation limit.

We observe the relation between survival rates at the critical point and k-shell indices, excluding small number shells to ignore the observation limit. Fig 10(c) represents the median of *P*_*s*_(*f*_*c*_) calculated for each shell. It is natural that the median survival rate increases for larger shell indices. We empirically obtain an exponential relationship described by the following equation, excluding the largest shell index, k = 25,

$$\begin{array}{c} P_{s}\left(f_{c}\right)=A\exp\left(Bk_{s}\right) \end{array}$$ (13)

where *A* = 1.7×10^−4^ and *B* = 0.16. This result indicates that the survival rate increases exp(B) = 1.17 times per shell index increment.

We can explain the non-trivial relation between the shell and survival rate in the following manner. We assume that the survival rate of a node *i*, *P*_*s*, *i*_, is simply written as the fraction of removed links *f*, as follows:

$$\begin{array}{c} P_{s,i}=1-f^{\bar{k_{i}}} \end{array}$$ (14)

where $\bar{k_{i}}$ is the number of links that contribute to the connectivity of the largest cluster. Eq (14) can be approximated as Eq (15) below because the value 1−*f* is very small at the critical point (*f*_c_ = 0.994). We take the average of *P*_*s*, *i*_ for each shell.

$$\begin{array}{c} P_{s,i}≃\bar{k_{i}}\left(1-f\right) \end{array}$$ (15)

$$\begin{array}{c} P_{s}≃{\left\langle \bar{k}\right\rangle }_{s}\left(1-f\right) \end{array}$$ (16)

Here, ⟨⋅⟩_*s*_ denotes the average survival rate of nodes in each shell and *s* is the index of the shell. Accordingly, we determine the slope of Fig 10(c) by the following equation:

$$\begin{array}{c} B∼\frac{1}{s_{\max}-s_{\min}}\sum_{s_{\min}}^{s_{\max}-1}\log\frac{{\left\langle \bar{k}\right\rangle }_{s+1}}{{\left\langle \bar{k}\right\rangle }_{s}}=\frac{1}{s_{\max}-s_{\min}}\log\frac{{\left\langle \bar{k}\right\rangle }_{s_{\max}}}{{\left\langle \bar{k}\right\rangle }_{s_{\min}}} \end{array}$$ (17)

We then evaluate $\bar{k_{i}}$, which are links whose removal causes the connectivity of the largest cluster to decrease. We introduce a new decomposition of link number, *k*, of a node into the following three numbers:

$$\begin{array}{c} k=k_{u}+k_{m}+k_{d} \end{array}$$ (18)

where *k*_*u*_ is the number of links connected to an upper shell, *k*_*m*_ is the number of links linked to the same shell, and *k*_*d*_ is the number of links that connect the node to lower numbered shells, as schematically shown in Fig 10(d). We believe that *k*_*u*_+*k*_*m*_ is $\bar{k_{i}}$ because it is likely that a connection to lower numbered shells will not increase the probability of membership in the largest cluster as the largest cluster is generally composed of nodes with higher shell indices.

$$\begin{array}{c} \bar{k_{i}}=k_{u}+k_{m} \end{array}$$ (19)

From the network data we can calculate the mean value for each shell, ${\left\langle \bar{k}\right\rangle }_{s}$, by the following equation:

$$\begin{array}{c} {\left\langle \bar{k}\right\rangle }_{s}={\left\langle k_{u}+k_{m}\right\rangle }_{s} \end{array}$$ (20)

Using the results obtained from Eq (17), we obtain *B* ∼ 0.160, which agrees very well with the empirical slope of Fig 10(c).

Note that the median survival rate for the shell with the highest index does not fit well with Eq (13). We consider this discrepancy to be caused by the fact that *k*_*u*_ in the shell with highest index is 0 in Eq (18), and it is likely that the shell has a hierarchical structure that includes deeper cores of nodes with larger survival rates. This situation implies that the survival rate at the critical point can be a new quantitative characterization of the importance of a node, which is roughly proportional to the shell index but can differ among nodes in the same shell index.

**Fig 11:**
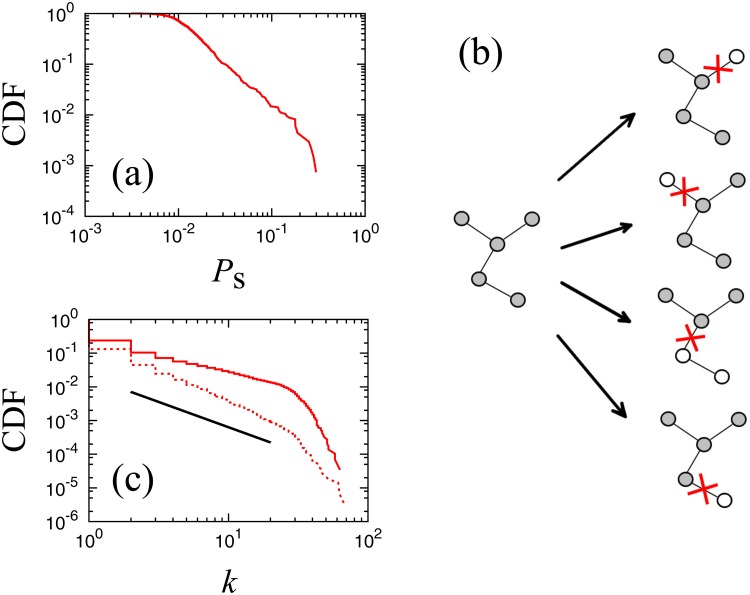
**(a) Cumulative distributions of the survival rate at the critical point (*f*_c_ = 0.994) of nodes belonging to the largest shell, *k*_*s*_ = 25, in the initial state. (b) Schematic figure of calculating the survival rate**. Each link is supposed to be removed with the same probability and we compare the sizes of separated clusters. The gray nodes belong to the largest cluster. **(c) Cumulative distribution of link numbers at the critical point in a log-log plot**. The solid line is calculated only in the largest cluster, and a superposition of 100 trials. The dotted line is calculated for all clusters, and we take superposition of 10 trials. The guide line shows the slope of 1.5, the same slope as Fig 1(a).

Fig 11(a) shows the distribution of the survival rate of nodes belonging to the 25-shell. This figure suggests that the survival rate of the nodes in this highest shell has wide diversity. In order to understand this diversity, for comparison, we theoretically calculate the survival rate for a Cayley tree, which is a regular tree network with link number *K* for all nodes, a typical theoretical model of a loopless network. We remove a link as schematically shown in Fig 11(b) and calculate the probability of a node belongings to the largest cluster. Although we will not derive it here, we can show that the survival rate distribution for a Cayley tree of the total link number *M* is given as, *f*(*P*_*s*_) = (*k*(*k*−1)/(*M*+1))exp(−*MP*_*s*_+*M*−1), an exponential distribution, which is much less diverse than the real survival rate distribution. As discussed in Section 5, the critical clusters, as with those of a Caley tree, are almost loopless; the difference in the survival rate distribution may be caused by the non-uniformity of the link numbers. We can directly observe non-uniformity of link numbers of nodes in the critical clusters as shown in Fig 11(c). We can confirm that the slope of the distribution is smaller for the critical clusters than for the original network shown in Fig 1(a), implying that the link number distribution is highly non-uniform; this result may be a cause for the wide diversity in the survival rate.

The node with the largest survival rate has 6,449 links, which is the second highest in the link number ranking. The node with the largest number of links, however, is 36 th in the survival rate ranking. This result shows that the link number is not proportional to the survival rate. The survival rate of a node at criticality can provide new information about the importance of the node.

Based on survival rate ranking, we select the top 100 firms and observe their job categories. The top job category is manufacturing that captures 48% share while it is 25% in the original network, and the second category is construction that captures 28% share while it is 21% in the original network. These results suggest that manufacturing and construction businesses play a more important role in the business network than other industries. This method statistically shows robust job categories.

## Conclusion

In this paper, we analyzed the link-removal percolation transition of a complex business relation network through precise numerical calculation, and concluded that the critical exponents are given by mean-field values. This result occurs because a number of loop links vanish at the same critical point, and the mean-field approximation becomes exact. We also discussed the finite size dependency of this property, and confirmed the agreement between observation and theory. For ER-graphs, we showed that the finite size scaling relations are consistent with the ER-graph percolation theory based on the mean link number per node being 1 at the critical point. We argued that this type of sparse graph limit transition is observable in general complex networks whose mean link number per node tends to diverge.

Note that the above discussion is applied for networks with *ϕ* > 1. In the case of *ϕ* = 1 such as two dimensional regular lattices, the transition point can take a value between 0 and 1 in the infinite scale limit. If we modify the condition of random removal to some designed ways of removal such as targeted attacks for links connecting with higher degree nodes, there is a possibility of realizing percolation transition with a non-trivial transition point, *f*_*c*_, even in the case of *ϕ* > 1.

We introduced the survival rate as a new index characterizing the robustness of nodes, and showed that this value is closely related to the index of k-shell decomposition analysis. In addition, we confirmed that the survival rates distribute widely for the same *k*-shell nodes; also, their values are not simply proportional to the link numbers. Therefore, the survival rates estimated at the percolation critical point can be an independent measure for nodes, representing robustness for random failure of a complex network. Using real-world data, we found that businesses categorized in construction and manufacturing tend to have larger survival rates in a network than other job categories.
